# Electroacupuncture improves neurocognitive impairment induced by chronic sleep deprivation: an experimental study based on hippocampal neurogenesis and synaptic plasticity

**DOI:** 10.3389/fnhum.2026.1857669

**Published:** 2026-07-31

**Authors:** Jianli Wu, Jiajing Sun, Mengdi Li, Hao Wang, Geling Lu, Chen Guo, Kunping Jia, Miao Zhang

**Affiliations:** 1 Institute of Traditional Chinese Medicine, Heilongjiang University of Chinese Medicine, Harbin, Heilongjiang, China; 2 Department of Acupuncture, The First Affiliated Hospital of Heilongjiang University of Chinese Medicine, Harbin, Heilongjiang, China; 3 Department of Acupuncture, The Second Affiliated Hospital of Heilongjiang University of Chinese Medicine, Harbin, Heilongjiang, China

**Keywords:** chronic sleep deprivation, cognitive impairment, electroacupuncture, hippocampal neurogenesis, synaptic plasticity

## Abstract

**Objective:**

Normal sleep rhythms are crucial for hippocampus-dependent advanced cognitive functions. Chronic sleep deprivation (CSD) impairs hippocampal neurogenesis and structure, leading to neurocognitive deficits. Electro-nape-acupuncture (ENA) at bilateral Fengchi (GB20) and Gongxue (Extra) is a specialized acupuncture technique for treating insomnia, amnesia, and other brain-originated diseases. This study aims to investigate whether ENA improves CSD-induced cognitive impairment by regulating neurogenesis and synaptic plasticity in the hippocampus.

**Methods:**

The modified multi-platform water environment method was used to establish the CSD model. Electroacupuncture or sham electroacupuncture was used to treat bilateral cervical acupoints (Fengchi and Gongxue) for 20 min, once a day for 14 days. The Morris water maze experiment evaluated spatial learning and memory in rats, and the new object recognition experiment evaluated recognition memory. Immunofluorescence (IF) staining and Western Blot (WB) were used to detect the expression levels of the hippocampal neurogenesis markers, doublecortin (DCX) and Ki-67. Golgi-Cox staining and transmission electron microscopy were used to observe the changes in neurons and synaptic plasticity in the dentate gyrus (DG) of the hippocampus.

**Results:**

Neurocognitive impairment induced by CSD is associated with abnormal changes in hippocampal neurogenesis and synaptic plasticity. The results of IF and WB showed that the protein expressions of DCX and Ki-67 in the hippocampus of CSD rats were significantly decreased. Transmission electron microscopy revealed that in the DG region of the hippocampus of CSD rats, the synaptic density and the thickness of the postsynaptic density membrane decreased, while the synaptic cleft width increased. Golgi staining showed that the density of dendritic spines in the DG area of the hippocampus in CSD rats decreased significantly, especially mushroom-shaped dendritic spines. ENA can enhance spatial learning and memory, as well as recognition memory, induced by CSD, and effectively reverse the abnormal changes in neurogenesis and synaptic plasticity in the DG region of the hippocampus.

**Conclusion:**

In male Wistar rats, ENA improves neurocognitive function by promoting neurogenesis in the hippocampal dentate gyrus and restoring synaptic plasticity, thereby reconstructing neural memory circuits. ENA therapy offers a new strategy for treating cognitive impairments related to chronic sleep deprivation in males, and holds potential significance for the clinical management of cognitive impairment diseases.

## Introduction

Insufficient sleep has emerged as a widespread public health issue, driven by factors such as social stress, environmental pollution, and various chronic diseases (Grandner, 2022; Cao et al., 2021; Yang et al., 2018). Chronic sleep deprivation (CSD) disrupts natural circadian rhythms, reduces total sleep duration, impairs cognitive performance, and exacerbates anxiety and depressive symptoms-a phenomenon particularly prevalent among shift workers (Casas-Méndez et al., 2026; Zhang et al., 2026). It has been reported that approximately 20% of adults worldwide have insomnia, and the persistence rate within 5 years is as high as 40% (Morin and Jarrin, 2022). Notably, average sleep duration has declined significantly over the past few decades (Ford et al., 2015; Groeger et al., 2004). Given the critical role of sleep in maintaining cognitive functions, this trend is a source of concern. Sleep deprivation impairs multiple cognitive domains, including attention, decision-making, and memory, ultimately compromising neurocognitive function (Eichenlaub et al., 2020). Furthermore, long-term sleep deficiency may hinder the clearance of beta-amyloid, thereby accelerating the pathological progression of Alzheimer’s disease (Zhao et al., 2019; Chen et al., 2017). A recent cohort study involving 868 participants further indicated that each 1% reduction in slow-wave sleep is associated with a 27% increase in dementia risk (Himali et al., 2023). While conventional pharmacological treatments for cognitive impairment have been extensively studied, they have yet to yield transformative outcomes. Available medications often provide only symptomatic relief and are frequently associated with adverse effects (Pérez Palmer et al., 2022). In light of these limitations, non-pharmacological interventions such as acupuncture have attracted growing interest as potential approaches to restore cognitive function with improved safety profiles.

Growing evidence indicates that acupoint stimulation has a significant effect on cognitive function. For instance, a recent clinical study reported that stimulation of the Shenting (GV24) and Baihui (GV20) acupoints reduced the incidence of postoperative cognitive dysfunction (Zheng et al., 2025). Similarly, functional MRI studies have shown that acupuncture at Hegu (LI4) and Taichong (LR3) modulates functional connectivity between the retrosplenial cortex and the hippocampus in patients with Alzheimer’s disease (Wang et al., 2024). Shenmen (HT7) and Zusanli (ST36) also influenced brain regions involved in spatial vision and cognition (Hu et al., 2025). These findings indicate that acupuncture positively affects cognitive functions by regulating multiple brain regions involved in memory. It has been confirmed that electrical stimulation can regulate synaptic plasticity and enhance cognitive function (Simonsmeier et al., 2018). Building on this foundation, electro-nape-acupuncture (ENA) at bilateral Fengchi (GB20) and Gongxue (Extra) a specialized technique developed by Professor Gao Weibin for brain-derived disorders—has demonstrated notable clinical benefits in addressing insomnia and its frequently comorbid symptoms, such as anxiety, depression, and memory decline. By integrating electrical stimulation with acupuncture at specific neck acupoints, ENA offers a dual-therapy approach that may synergistically promote neurocognitive recovery. Our group’s previous clinical research has demonstrated that electroacupuncture at Fengchi (GB20) and Gongxue (Extra) can improve memory function in patients with chronic insomnia. Moreover, animal studies have shown that electroacupuncture can alleviate neuronal swelling in the hippocampal dentate gyrus (DG) of rats with chronic sleep deprivation, increase the number of granule cells, and facilitate neuronal repair (Wang et al., 2021). Nevertheless, the mechanisms underlying ENA’s neuroprotective effects on hippocampal neurons remain unclear.

The hippocampus plays a vital role in memory encoding and storage, and impaired hippocampal neurogenesis is a recognized pathogenic mechanism in cognitive disorders (Pronier et al., 2023; Vivar, 2015). CSD is known to disrupt the hippocampal microenvironment by inducing oxidative stress, neuroinflammation, and protein aggregation, ultimately leading to suppressed neurogenesis (Atrooz and Salim, 2020; Wang and Holtzman, 2020). Structural and cellular changes evidence this suppression; for instance, magnetic resonance imaging has revealed significantly reduced bilateral hippocampal volume in patients with insomnia, and experimental studies have confirmed that CSD inhibits the proliferation and differentiation of neural stem cells in the dentate gyrus, thereby impairing memory (Chen et al., 2022; Shixing et al., 2023). While acupuncture has demonstrated clinical benefits for cognitive decline, the specific mechanism of ENA therapy in this context is not fully elucidated. A critical gap remains in understanding whether ENA’s efficacy against CSD-induced impairment is mediated by promoting hippocampal neurogenesis. Therefore, we hypothesize that ENA mitigates cognitive dysfunction by enhancing hippocampal neurogenesis and synaptic plasticity. The process of hippocampal neurogenesis will be evaluated by the increased expression of the canonical markers Ki-67 and DCX.

Therefore, this study established a CSD model using the modified multi-platform water environment method. We aimed to elucidate the mechanism by which ENA improves CSD-related cognitive impairment by observing changes in DG neurons and synaptic plasticity and by detecting the expression of neurogenesis-related molecules, including doublecortin (DCX) and Ki-67. Our findings may open new avenues for treating CSD-induced cognitive disorders and provide a scientific basis for the use of acupuncture in cognitive restoration. Furthermore, by clarifying the role of ENA in hippocampal neurogenesis, this study offers novel insights into non-pharmacological interventions for sleep-related cognitive decline.

## Materials and methods

### Antibodies and reagents

The following are the primary reagents used in this study: anti-DCX (1:1000, 13,925-1-AP, Proteintech); anti-Ki-67 (1:500, 28,074-1-AP, Proteintech); anti-GAPDH (1:10000, 10,494-1-AP, Proteintech); 488-Goat Anti-Rabbit Secondary Antibody (1:200, RGAR002, Proteintech). BCA protein assay kit (P0012, Beyotime); SDS-PAGE (P0690, Beyotime); RIPA buffer (P0013C, Beyotime); PBS buffer solution (C0221A, Beyotime); phosphatase inhibitors (04906837001, Roche); Golgi staining kit (PK401, FD NeuroTechnologies); 4% polyformaldehyde (BL539A, Biosharp); Glutaraldehyde (TED PELLA, 2171220).

### Animals and grouping

A total of 60 male adult specific pathogen-free Wistar rats (age 6–8 weeks, weighing 180–220 g) were used in this study. The animals were supplied by the Experimental Animal Center of Heilongjiang University of Chinese Medicine [License No. SCXK(Hei)2023–001]. All rats were housed under controlled conditions: room temperature of 23 ± 1 °C, relative humidity of 60 ± 5%, and a 12-h light/dark cycle with lights on at 7:00 (Zeitgeber Time 0, ZT0) and off at 19:00 (ZT12) (Agamme et al., 2024). Food and water were available ad libitum throughout the study.

After 1 week of acclimatization, 12 rats were randomly assigned to the tank control (TC) group. The remaining animals underwent a CSD protocol to establish the model. Thirty-six successfully modeled rats were then randomly allocated into three groups (*N* = 12 per group): the model (Mod) group, the ENA group, and the Sham ENA group. The entire experimental protocol was approved by the Animal Ethics Committee of Heilongjiang University of Chinese Medicine (Ethical Approval Code: 2024042601). All procedures were conducted in strict compliance with the Chinese Regulations for the Administration of Affairs Concerning Experimental Animals (2017) and the U. S. National Research Council’s Guide for the Care and Use of Laboratory Animals (Eighth Edition). Furthermore, this study adhered strictly to the ARRIVE guidelines to ensure methodological rigor and ethical reporting. All personnel involved in animal handling, anesthesia, and euthanasia were rigorously trained to ensure proficiency (Kilkenny et al., 2012).

Animal welfare was safeguarded through twice-daily monitoring, with a body weight loss exceeding 20% from baseline set as a stringent humane endpoint criterion. No animals met this criterion during the study. Upon termination, all rats were humanely euthanized via an intraperitoneal overdose of pentobarbital sodium (100 mg/kg), followed by cervical dislocation as a secondary measure to ensure death.

### CSD procedure

**Figure 1:**
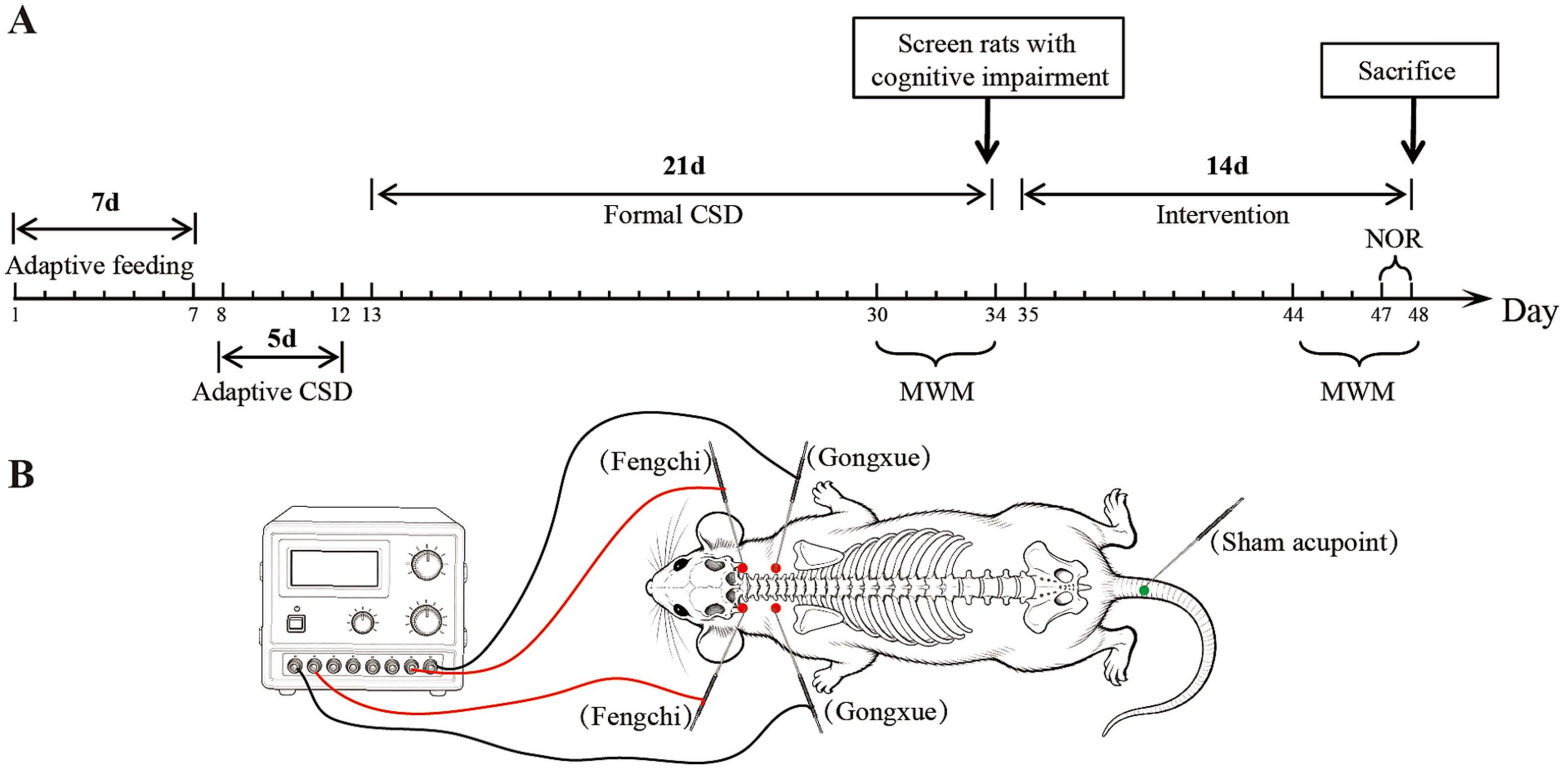
Experimental timeline and acupoint locations. **(A)** Experimental flow chart. The experimental procedure included acclimatization (days 1–7) with adaptive CSD (days 8–12), followed by 21 days of formal CSD (days 13–34). After CSD, the first MWM test established a baseline (days 30–34). Rats then underwent a 14-day intervention (days 35–48), after which the MWM and NOR tests were repeated (days 44–48). All animals were sacrificed after the behavioral testing concluded on day 48. Animal identification remained consistent throughout the study. **(B)** Schematic diagram of acupoints locations.

The CSD model was induced using a modified multi-platform method (MMPM) over 21 days (Cao et al., 2019; Yin et al., 2024). Briefly, the procedure was conducted in a custom-made transparent water tank (90 × 70 × 50 cm) containing 12 round platforms (diameter: 6.5 cm; height: 8 cm). The platforms were arranged with a center-to-center distance of 20 cm to allow free movement. The water level was maintained 1 cm below the platform surface, forcing the animals to remain awake on the small, unstable surfaces. Before the formal 21-day CSD protocol, all rats underwent a 5-day adaptation phase, with daily sleep deprivation from ZT9 (16:00) to ZT11 (18:00). The formal CSD regimen consisted of 18 h of sleep deprivation (from ZT9 to ZT3 the following day) followed by 6 h of sleep recovery, repeated for 21 cycles. During deprivation, the environment was illuminated, and food and water were accessible through wire-mesh covers placed above the platforms. To control for the stress of the water environment, rats in the TC group were housed in an identical tank lined with a continuous wire-mesh floor, allowing for normal sleep (Figure 1A).

Following the 21-day modeling period, we screened rats with cognitive impairment using the Morris water maze test. Inclusion criteria are as follows: only CSD rats with an escape latency exceeding the TC group’s average by more than 20% were deemed successfully impaired and included in subsequent experiments.

### Intervention treatment

The rats in both the TC and Mod groups underwent the same restraint procedure for the same duration but received no acupuncture stimulation.

After modeling was completed, rats in the ENA group were treated with ENA at the bilateral Fengchi (GB20) and Gongxue (Extra) points for 20 min daily from ZT1 to ZT3 for 14 days. Before treatment, animals were secured on a custom-designed platform to keep their heads stationary. Then the neck hair was shaved off, and the skin was disinfected. Bilateral acupoints were selected: FengChi (GB20), located 2 mm lateral to the depression at the occipital condyle, and GongXue (Extra), situated 0.5 cm directly below FengChi (approximately 2 mm lateral to the spinous process of the fourth cervical vertebra). Acupoint localization was based on established rat acupuncture maps and comparative neuroanatomy (Xu et al., 2019; Zhu et al., 2023). The 0.18 × 25 mm sterile needles were inserted 4–5 mm toward the nose tip (Figure 1B). The parameters of the electroacupuncture device were set to sparse-wave, 2 Hz, and 0.5 mA, based on previous relevant studies (Wu et al., 2021). The rats in the Sham ENA group were stimulated at the tail root proximal to the trunk, using the same method as in the ENA group, but without electrical stimulation.

### Behavioral experiment

#### Morris water maze test

The rats’ spatial learning and memory were assessed using the Morris water maze (MWM) test. The protocol consisted of two identical training-and-testing cycles, conducted from ZT3 to ZT5 on days 30–34 and 44–48 of the experiment, respectively (Vivar, 2015). Training Phase (Acquisition): For each cycle, rats underwent four consecutive days of training. Each day, they performed 4 trials, starting from each of the 4 quadrants in a randomized order, with a 30-min inter-trial interval. The goal was to locate a hidden platform submerged in quadrant III. If a rat failed to find the platform within 90 s, it was gently guided to it and allowed to remain there for 10 s. Testing phage (Memory Retention): On the day following the final training session (Days 34 and 48), positioning cruise and spatial exploration tests will be conducted. The time taken for rats to navigate from the starting quadrant I to the submerged platform is recorded as the escape latency. The submerged platform was then removed, and each rat was allowed to swim freely for 120 s. The number of crossings over the former platform location and the time spent in the target quadrant (where the platform was previously located) were recorded and analyzed.

#### New object recognition experiment

The novel object recognition (NOR) test was conducted over three consecutive stages: adaptation, training, and testing. All sessions were performed in an open-field test box. Adaptation phase (Day 47): Rats were transported to the testing room and allowed to acclimate for 30 min in both the afternoon (from ZT5 to ZT6) and evening (from ZT11 to ZT12) sessions. Subsequently, each rat was placed individually in the empty test box and allowed to explore freely for 5 min before being returned to its home cage. Training phase (Afternoon of Day 48): Two identical objects (Object A) were positioned symmetrically, approximately 10 cm from the walls of the test box. Each rat was then introduced to the box and allowed to explore the two identical objects for 5 min freely. Testing phase (Evening of Day 48): One of the familiar Object A items was replaced with a novel object (Object B). The rat was returned to the box, and its exploration time directed toward Object A (familiar) and Object B (novel) was recorded over 5 min. A recognition index was calculated as follows: Recognition Index = Time exploring Object B/(Time exploring Object A + Time exploring Object B). To eliminate olfactory cues, the test box and objects were thoroughly cleaned with 75% ethanol after each rat completed the session (Grayson et al., 2015).

### Molecular detection

#### Immunofluorescence

The IF assay was used to determine the effect of the ENA’s on the fluorescent expression of Ki-67 and DCX in the DG region of the hippocampus. The paraffin-embedded tissue was cut into 5 μm sections, dewaxed, hydrated, antigen-repaired, and sealed. Ki-67 (1:400) and DCX (1:500) primary antibodies were added separately and incubated for 1 h at room temperature in a humidified box. Then, the fluorescent secondary antibody (1:200) was incubated for 1 h at room temperature. DAPI-stained nuclei for 20 min, then added a fluorescent quencher. Photographing with a fluorescence microscope: Take three non-overlapping photographs at 400 times, and save them in JPG format. Use the ImageJ software to analyze the number of positive cells and the average fluorescence intensities of DCX and Ki-67 in the DG region.

#### Western blot

The protein expression of Ki-67 and DCX in the right hippocampal tissue was analyzed by Western blot. Briefly, tissues were homogenized in RIPA buffer containing protease inhibitors at a 1:10 (w/v) ratio. The total protein concentration of each sample was determined using a BCA assay kit according to the manufacturer’s instructions. Subsequently, protein samples were mixed with loading buffer, boiled at 100 °C for 5 min, and cooled to room temperature. After separation by SDS-PAGE and transfer to PVDF membranes, the membranes were blocked and then incubated overnight at 4 °C with primary antibodies against Ki-67 (1:500) and DCX (1:1000). Following washing, the membranes were incubated with horseradish peroxidase-conjugated secondary antibodies (1:10,000) for 45 min at room temperature. Protein bands were visualized using an ECL substrate and imaged with a gel documentation system. The band intensities were quantified in ImageJ, and the relative expression of each target protein was calculated.

### Transmission electron microscopy

TEM was performed in the DG region of hippocampal tissue to clarify the ENA’s effects on neuronal and synaptic structures. The hippocampal tissue was fixed in osmium acid, dehydrated in acetone, and embedded in epoxy resin. Subsequently, it was cut into 70 nm ultrathin sections and stained with uranyl acetate and lead citrate. Under a TEM, we can observe structural changes in the nuclear membrane, chromatin, mitochondria, endoplasmic reticulum, synapses, and other neuronal structures. Three non-overlapping images of each sample were taken at 5,000 × magnification. Two researchers counted synapses under the electron microscope and calculated synapse density.

### Golgi-Cox staining

The morphological alterations and quantitative changes in dendritic spines within the DG region of the rat hippocampus were evaluated using Golgi staining. Fresh brain tissues were washed with PBS and fixed in 4% paraformaldehyde for 24 h. Then, the specimens were cut into three coronal sections and kept in Golgi staining solution at room temperature in the dark for 14 days, followed by storage in 30% sucrose solution at 4 °C in the dark for 3 days. The coronal sections were cut into 100 μm slices, stained with Golgi’s solution, dehydrated through a graded ethanol series, rinsed with xylene, sealed with resin glue, and then observed under a microscope. The density and morphological changes of dendritic spines were analyzed using the ImageJ software.

### Statistical analysis

All data were analyzed using SPSS software (version 24.0) and are presented as the mean ± standard deviation (SD). For comparisons across multiple groups, the normality of data distribution was first assessed. Data conforming to a normal distribution were analyzed by one-way analysis of variance (ANOVA), followed by *post hoc* tests for pairwise comparisons. If homogeneity of variance was confirmed, the Tukey’s test was applied; otherwise, Tamhane’s T2 test was used. For data that did not fit a normal distribution, the non-parametric Kruskal-Wallis H test was employed. The test level for the above statistical tests was *α* = 0.05, and differences were considered statistically significant when *p* < 0.05.

## Results

### ENA improves the spatial learning and memory abilities of CSD rats

**Figure 2:**
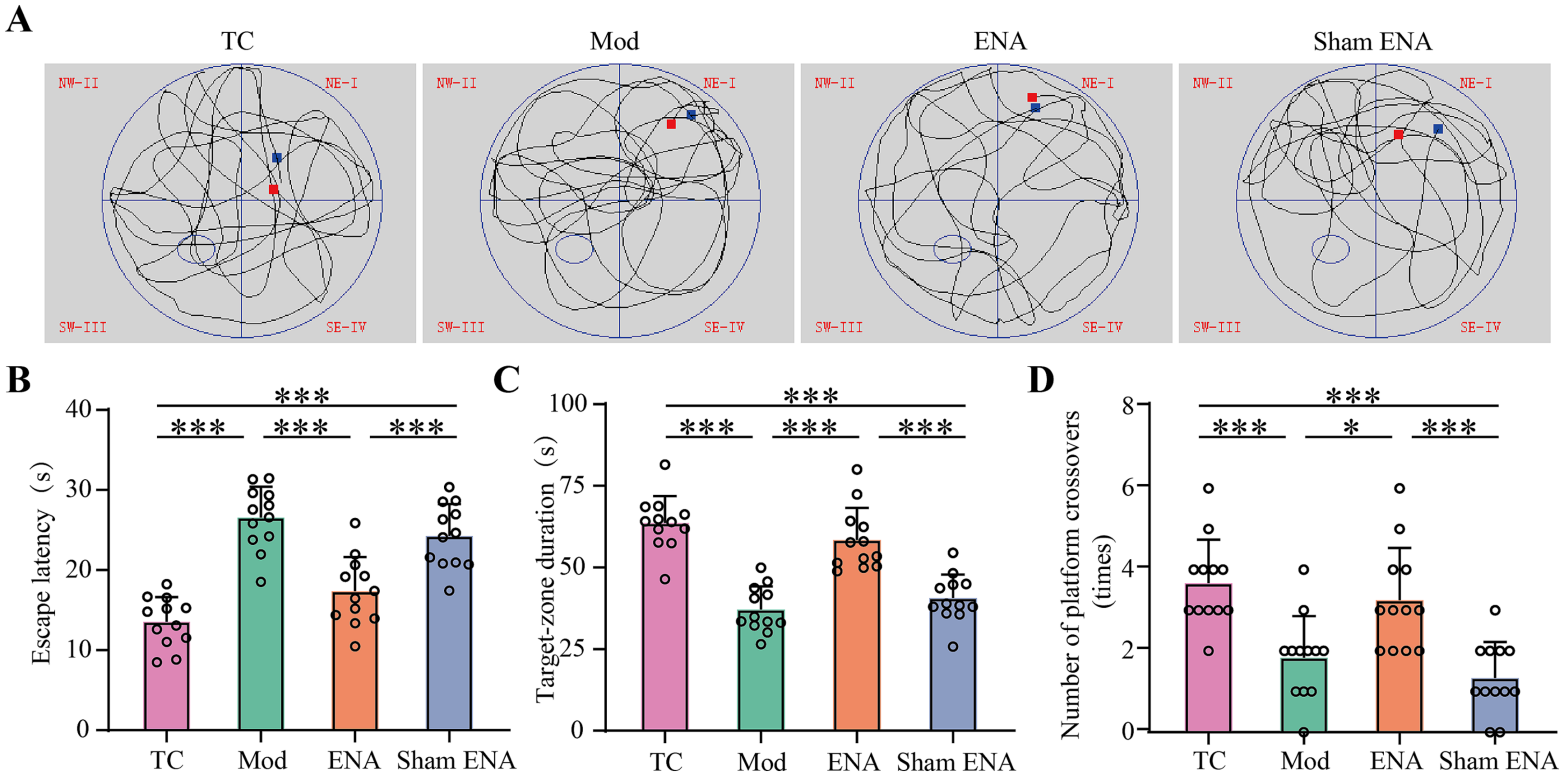
ENA improved the spatial learning and memory ability in CSD rats. **(A)** The typical trajectories of rats in each group crossing the original platform quadrant in the water maze space exploration experiment after treatment. **(B)** Escape latency. **(C)** Target-zone duration. **(D)** Platform crossing number. Data are presented as mean ± SD. *N* = 12 rats/group. Statistical significance indicated by ^*^p < 0.05, ^**^p < 0.01, ^***^p < 0.001, one-way ANOVA and Tukey’s test.

Representative swimming trajectories of each group in the MWM test are shown in Figure 2A. In the MWM test, compared with the TC group, rats in the Mod and Sham ENA groups exhibited significantly impaired spatial memory, as evidenced by prolonged escape latency (*F* = 29.54, *p* < 0.001; Figure 2B), reduced time spent in the target quadrant (*F* = 30.70, *p* < 0.001; Figure 2C), and fewer crossings over the original platform location (*F* = 12.80, *p* < 0.001; Figure 2D). The ENA group demonstrated significantly shorter escape latency, greater target-quadrant dwell time, and more platform crossings than the Mod group. The results indicated that ENA enhanced spatial learning and memory in CSD model rats.

### ENA improves non-spatial recognition memory in CSD rats

**Figure 3:**
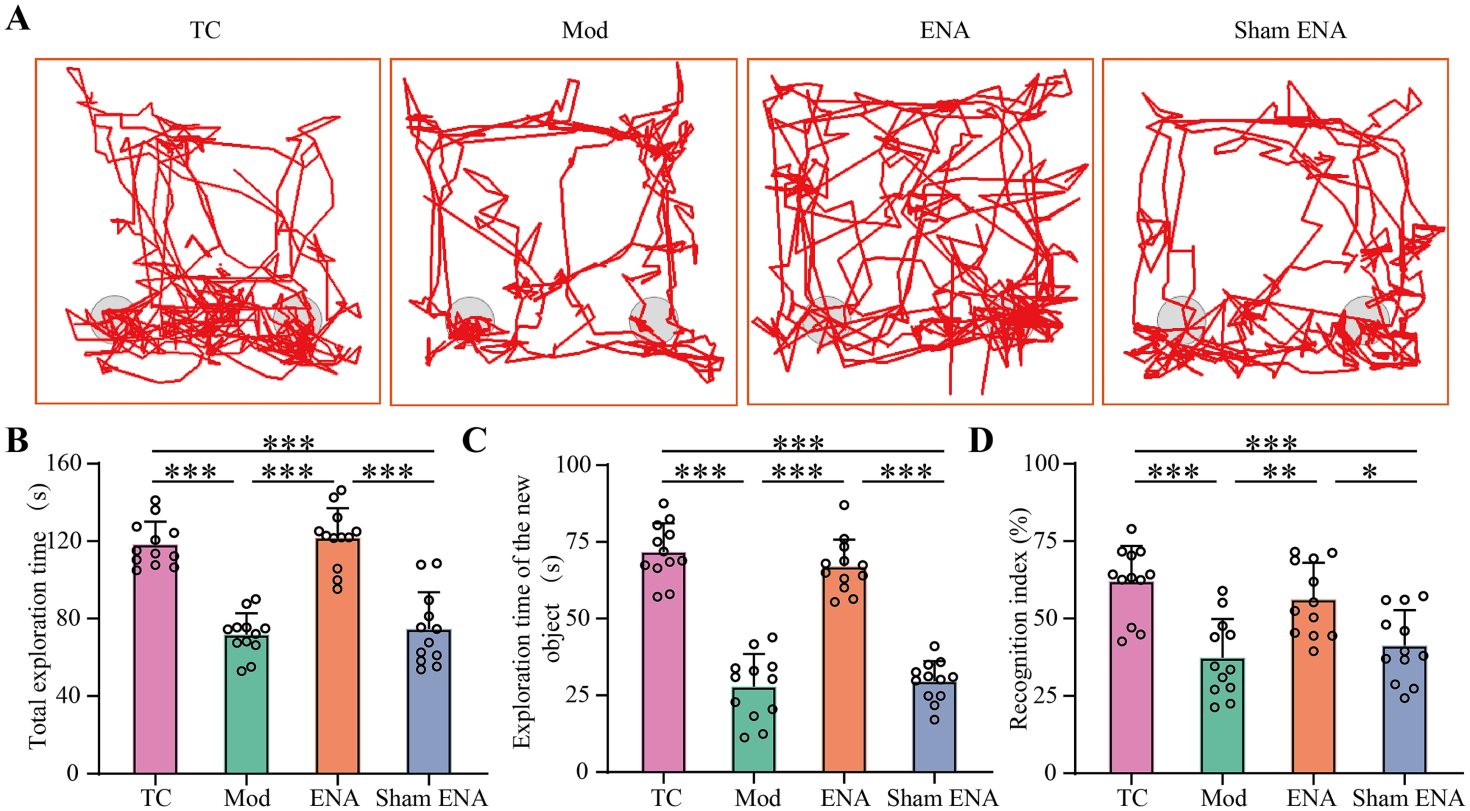
ENA improved non-spatial recognition memory ability in CSD rats. **(A)** The typical trajectories of rats in each group exploring objects in the test chamber after treatment (the left shade is a familiar object, and the right shade is a new object). **(B)** Total exploration time. **(C)** Exploration time of the new object. **(D)** Recognition index. Data are presented as mean ± SD. *N* = 12 rats/group. Statistical significance indicated by ^*^p < 0.05, ^**^p < 0.01, ^***^p < 0.001, one-way ANOVA and Tukey’s test.

Representative locomotor trajectories of each group in the NOR test are shown in Figure 3A. In the NOR test, rats in the Mod and Sham ENA groups exhibited significant recognition impairment compared to the TC group, as evidenced by reductions in total exploration time (*F* = 41.14, *p* < 0.001; Figure 3B), time spent exploring the novel object (*F* = 83.26, *p* < 0.001; Figure 3C), and the recognition index (*F* = 11.87, *p* < 0.001; Figure 3D) The ENA group showed significantly increased total exploration time, longer novel object exploration time, and a higher recognition index compared with the Mod group. The results indicated that ENA improves the non-spatial recognition memory abilities of CSD model rats.

### ENA increases the localization expression of Ki-67 and DCX in the DG region of the hippocampus of CSD rats

**Figure 4:**
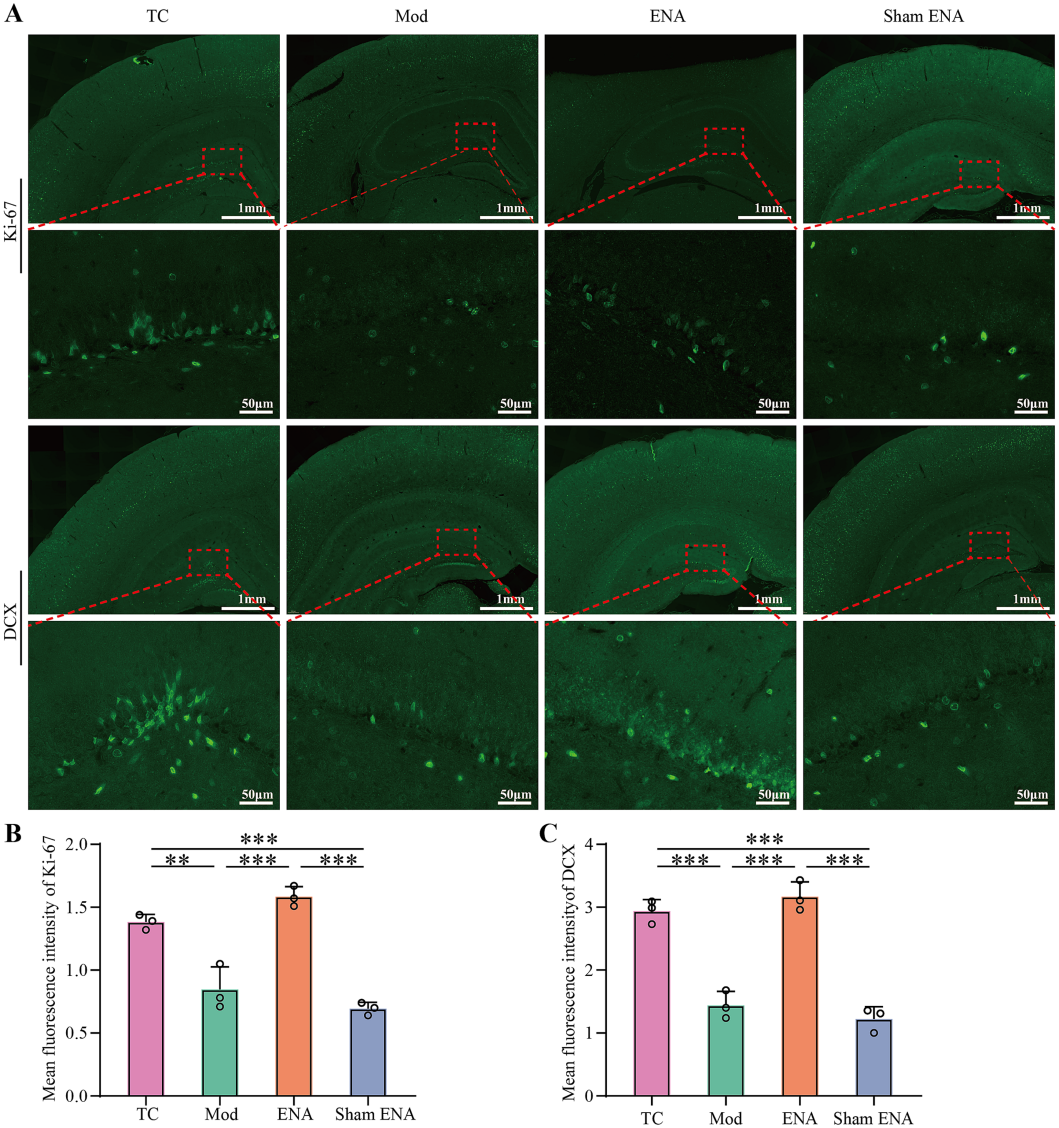
ENA upregulated Ki-67 and DCX fluorescence expression level in the DG region of the hippocampus in CSD rats. **(A)** Representative capture of Ki-67 and DCX immunofluorescence staining in the DG region of the hippocampus. **(B)** Mean fluorescence intensity of Ki-67. **(C)** Mean fluorescence intensity of DCX. Data are presented as mean ± SD. *N* = 3 rats/group. Statistical significance indicated by ^*^p < 0.05, ^**^p < 0.01, ^***^p < 0.001, one-way ANOVA and Tukey’s test.

Immunofluorescence analysis revealed significant differences in the content of neurogenesis markers in the DG region of the hippocampus among the groups (Figure 4A). Compared with the TC group, the average fluorescence intensities of Ki-67^+^ (*F* = 49.00, *p* < 0.001; Figure 4B) and DCX^+^ (*F* = 67.21, *p* < 0.001; Figure 4C) in the Mod and the Sham ENA groups were significantly decreased, indicating that the proliferation and differentiation functions of neurons in the DG area were inhibited after CSD. Compared with the Mod group, the average fluorescence intensities of Ki-67^+^ and DCX^+^ were increased, indicating that ENA treatment promoted the neurogenesis process in the DG region of the hippocampus.

### ENA increases the quantitative expression of Ki-67 and DCX in the hippocampus of CSD rats

**Figure 5:**
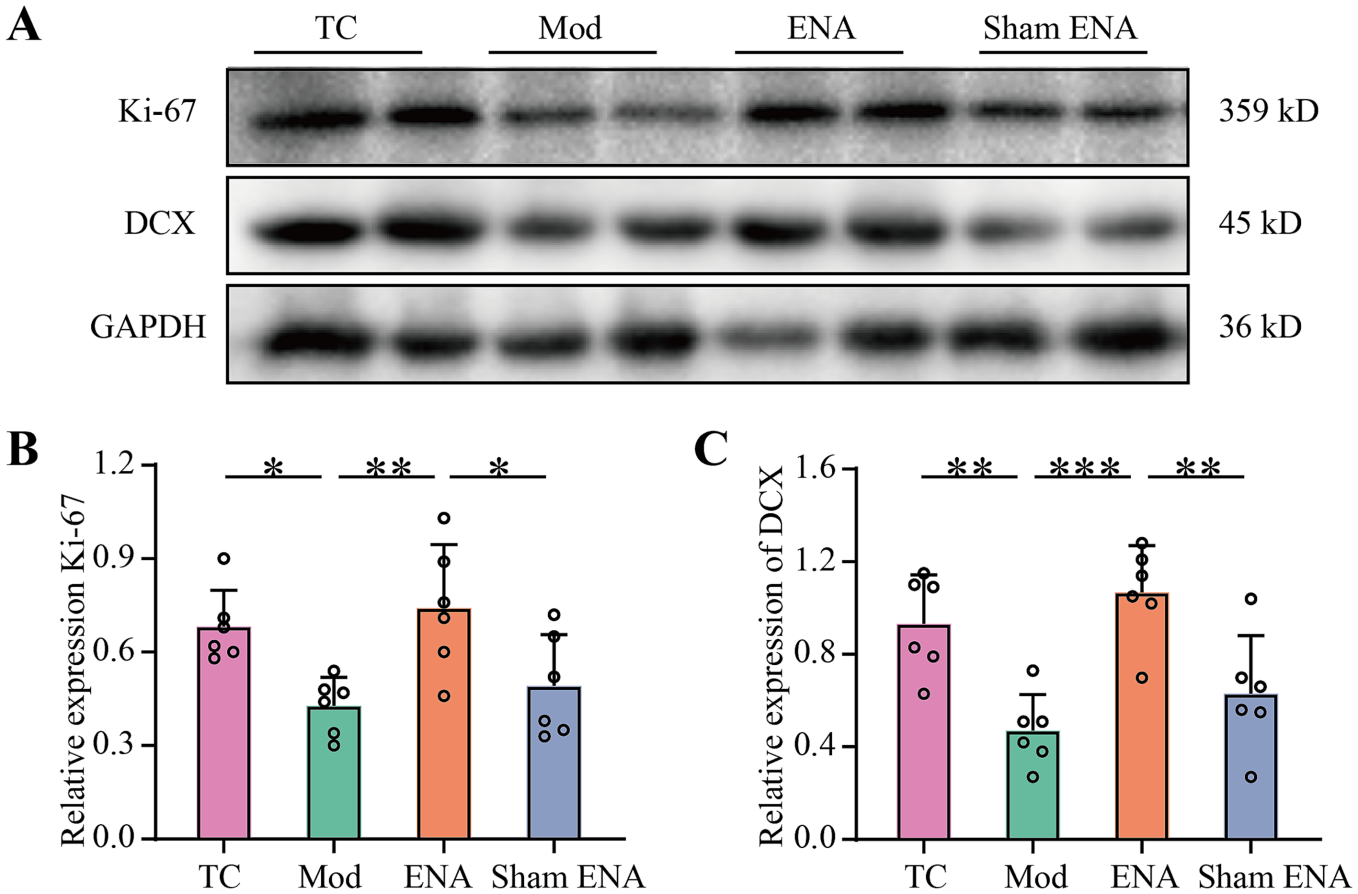
ENA activated the expression of Ki-67 and DCX in the hippocampus of CSD rats. **(A)** Western blot brands of Ki-67 and DCX in the hippocampus. **(B)** Relative expression of Ki-67. **(C)** Relative expression of DCX. Data are presented as mean ± SD. *N* = 6 rats/group. Statistical significance indicated by ^*^p < 0.05, ^**^p < 0.01, ^***^p < 0.001, one-way ANOVA and Tukey’s test.

Western blot analysis of hippocampal tissues yielded results consistent with the immunofluorescence findings (Figure 5A). Compared with the TC group, the relative protein expression levels of Ki-67 (*F* = 5.927, *p* = 0.005; Figure 5B) and DCX (DCX: *F* = 10.319, *p* < 0.001; Figure 5C) were significantly reduced in the Mod group. Compared with the model group, the relative protein expression levels of Ki-67 and DCX were significantly increased in the ENA group. ENA intervention can upregulate the quantitative expression of both neurogenesis markers and promote hippocampal neurogenesis in CSD rats.

### ENA promotes the repair of neurons of the DG region of the hippocampus in CSD rats

**Figure 6:**
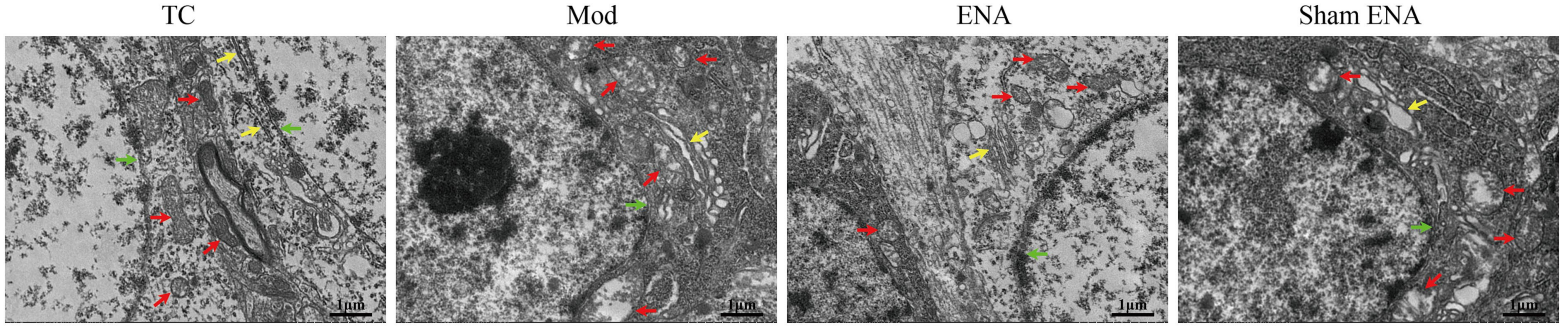
ENA attenuates neuronal damage in the DG region of the hippocampus in CSD rats. The green arrows point to the nuclear envelope, the red arrows point to the mitochondria, and the yellow arrows point to the endoplasmic reticulum. Scale bars = 1 μm.

Ultrastructural examination of hippocampal DG neurons in the TC group revealed normal morphology, characterized by round nuclei with intact membranes, uniformly distributed chromatin, and abundant organelles, including endoplasmic reticulum and mitochondria. The double-layered mitochondrial membrane structure was clear, and the mitochondrial cristae were neatly arranged. In contrast, neurons from the Mod and Sham ENA groups exhibited significant pathological changes, including cell shrinkage, nuclear membrane invagination, and clumped chromatin. Their cytoplasm contained swollen, vacuolated mitochondria with disrupted cristae, dilated endoplasmic reticulum, and an increased number of lysosomes. In the ENA group, neurons displayed more intact nuclear structure, granular chromatin, and mitochondria that were only mildly swollen with more preserved cristae (Figure 6). This demonstrates that ENA treatment can significantly attenuate neuronal damage in the DG region of the hippocampus.

### ENA restores synapse morphology and structure and increases the number of synapses of the DG region of the hippocampus in CSD rats

**Figure 7:**
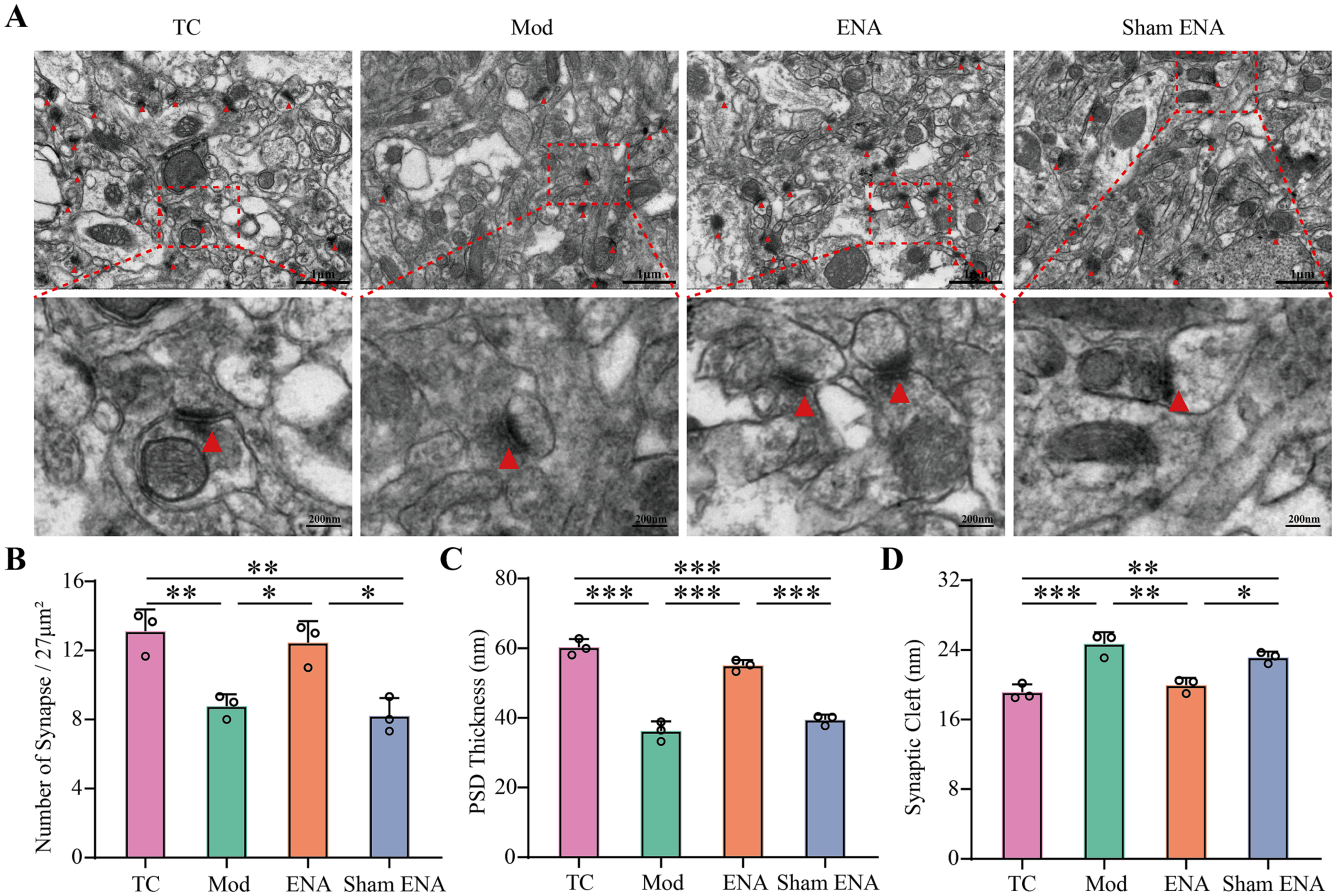
ENA repairing the morphology of the neurons and synapses in the DG region of the hippocampus in CSD rats. **(A)** The ultrastructure of the DG region of the hippocampus. The red triangle points to the synapse. **(B)** The number of synapses. **(C)** The thickness of postsynaptic density. **(D)** The width of the synaptic cleft. Data are presented as mean ± SD. *N* = 3 rats/group. Statistical significance indicated by ^*^p < 0.05, ^**^p < 0.01, ^***^p < 0.001, one-way ANOVA and Tukey’s test.

Ultrastructural analysis of the DG region revealed significant synaptic differences among the groups (Figure 7A). Compared with the TC group, the Mod and Sham ENA groups showed severe synaptic damage, with blurred synaptic structures, reduced synaptic vesicles, a significant decrease in the number of synapses (*F* = 15.88, *p* < 0.001; Figure 7B), a marked thinning of the postsynaptic density (*F* = 90.68, *p* < 0.001; Figure 7C), and widened synaptic clefts (*F* = 21.71, *p* < 0.001; Figure 7D). Compared with the model group, the ENA group showed less synaptic damage, clearer, more complete synaptic structures, increased synaptic vesicles, significantly more synapses, thicker postsynaptic densities, and narrower synaptic clefts.

### ENA enhances the density of dendritic spines and mushroom -shaped spines of the DG region of the hippocampus in CSD rats

**Figure 8:**
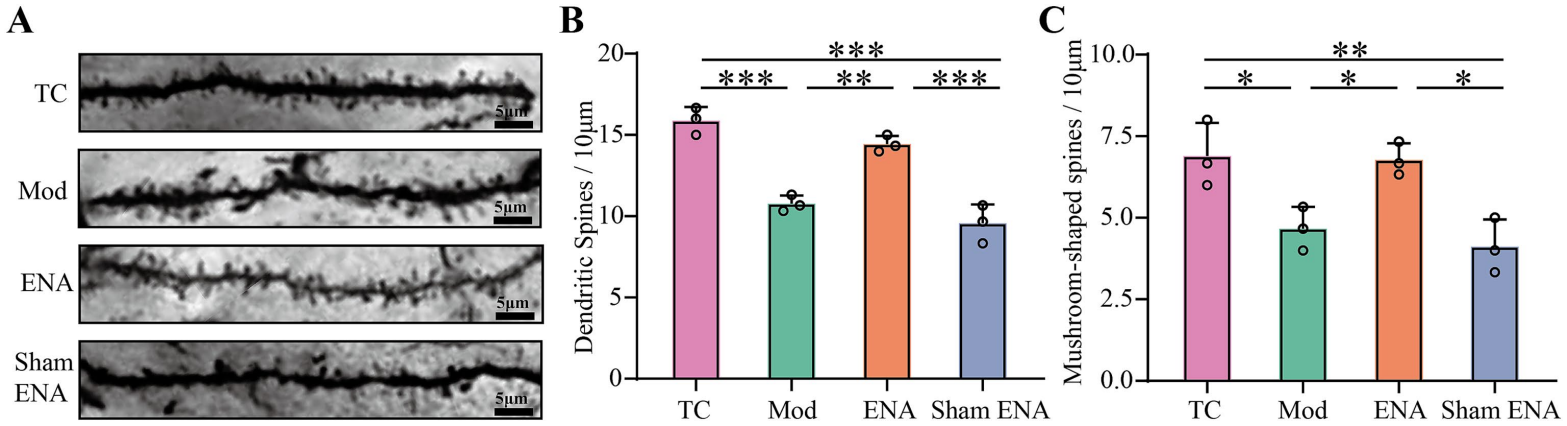
ENA enhances dendritic spine density in the DG region of the hippocampus in CSD rats. **(A)** Representative images of Golgi staining of dendritic spines in the granule cell layer of the hippocampal DG region. **(B)** Total dendritic spine density. **(C)** Density of mushroom-shaped dendritic spines. Data are presented as mean ± SD. *N* = 3 rats/group. Statistical significance indicated by ^*^p < 0.05, ^**^p < 0.01, ^***^p < 0.001, one-way ANOVA and Tukey’s test.

Golgi staining results showed that there were significant differences in the morphology of dendrites and the density of dendritic spines in the DG of the hippocampus among the groups (Figure 8A). In the TC group, dendritic spines predominantly exhibited mature morphological forms characterized as mushroom-like and stubby structures, which were observed in greater abundance. Compared with the TC group, the dendritic spines were in an immature state, presenting as slender or filopodia-like pseudopodia, and the total density of dendritic spines (*F* = 41.33, *p* < 0.001; Figure 8B) and mushroom-shaped dendritic spines (*F* = 10.04, *p* = 0.004; Figure 8C) decreased significantly in the Mod and Sham ENA. Compared with the Mod group, the dendritic spines were more mature, and the total density of dendritic spines and mushroom-shaped dendritic spines significantly increased in the ENA group. This suggests that ENA can alleviate the damage to dendritic spines and synaptic plasticity caused by CSD.

## Discussion

Sleep disorders are one of the important risk factors for cognitive impairment (Guarnieri and Sorbi, 2015; Borges et al., 2019). Acute insomnia can transiently disrupt cognitive domains such as attention, memory, and processing speed, deficits that are typically reversible upon sleep restoration (Hennecke et al., 2021). However, chronic sleep deprivation poses a more serious threat, leading to a progressive and insidious decline in higher-order cognitive functions, including executive function, complex memory, and social abilities (Khazaie et al., 2010). Critically, effective clinical strategies to reverse such cognitive damage remain elusive. Current pharmacological interventions, including neuroprotective and metabolic agents, often provide only temporary symptomatic relief, lack sustained efficacy after discontinuation, and can be cost-prohibitive (Montine et al., 2021; Massoud et al., 2007), underscoring the urgent need for alternative therapies.

Acupuncture is increasingly recognized as a potential therapy for brain-derived disorders. This study employed electroacupuncture at bilateral FengChi (GB20) and GongXue acupoints on the nape—a protocol derived from the clinical expertise of Professor Gao Weibin. Clinical observations suggest that this method, termed ENA, may ameliorate insomnia and its comorbid symptoms, including anxiety, depression, and memory loss (Wu et al., 2020). Fengchi is the intersection point of the Gallbladder Meridian and Yang Heel Vessel. It regulates the flow of gallbladder qi, calms the mind, and relieves fear. GongXue is a newly defined acupoint invented by Professor Gao, located 2 cm directly below FengChi on the human body. It is frequently used in combination for treating brain disorders such as insomnia, dizziness, forgetfulness, and stroke. The two acupoints are located on the nape of the neck, running along the Governing Vessel, the Bladder Meridian, and the Gallbladder Meridian from left to right. According to the theories that “all the yang meridians converge on the face” and “where the meridians pass, their therapeutic effects reach,” stimulating these two acupoints can unblock the three meridians, thereby exerting the functions of invigorating blood circulation, opening the orifices, refreshing the mind, and improving intelligence. Modern anatomical research has revealed that deep layers of the Fengchi and Gongxue acupoints are innervated by spinal sensory nerve fibers and supplied by the vertebral artery (Zheng et al., 2023). Stimulation of two acupoints can transmit signals from the posterior horn of the spinal cord to the reticular formation of the brainstem, thereby regulating the functions of the frontal lobe, hippocampus, and other brain regions. Animal studies have shown that electroacupuncture at the Fengchi point can improve learning and memory in rats (Lang et al., 2024). A clinical study also indicates that acupressure at the Fengchi point can effectively improve cognitive function and quality of life in older people (Lin et al., 2023). This study tested the spatial learning and memory abilities and the non-spatial recognition memory abilities of rats using the MWM and NOR tasks, respectively. The results were consistent with the above analysis. Compared with the TC group, the Mod group rats showed a significantly prolonged escape latency, fewer crossings over the original platform, and a decreased recognition index for new objects, indicating cognitive impairment after CSD. After 14 days of treatment, the escape latency of rats in the ENA group was significantly shortened, the number of crossings of the original platform position increased, the exploration time of new objects was prolonged, and the new object recognition index increased. However, there were no significant changes in the above-mentioned indicators in the Sham ENA group. Collectively, these behavioral findings confirm our initial hypothesis that ENA effectively ameliorates neurocognitive deficits induced by CSD.

Chronic sleep deprivation acts as a potent physiological stressor, primarily characterized by the activation of the hypothalamic–pituitary–adrenal (HPA) axis and subsequent dysregulation of stress hormones, including corticosterone (CORT) and adrenocorticotropic hormone. Elevated plasma CORT levels following sleep deprivation are well-documented (Tartar et al., 2009; Hairston et al., 2001). It is noteworthy that experimental procedures, such as restraint—a component of both modeling and acupuncture treatment—may also contribute to the overall stress burden. In our previous study (Wu et al., 2018), we confirmed that serum CORT levels were significantly elevated in sleep-deprived rats and that ENA intervention effectively downregulated these levels while promoting the restoration of normal sleep cycles. This finding suggests that ENA may confer therapeutic benefits by mitigating the aberrant HPA axis activation and stress response induced by CSD.

The pathogenesis of cognitive impairment associated with sleep disorders is complex, involving multiple factors, including oxidative stress, mitochondrial dysfunction, neuroinflammation, and abnormal neurogenesis (Musiek and Holtzman, 2016; Mukherjee et al., 2024). The DG of the hippocampus is a primary site of adult hippocampal neurogenesis (AHN), a process crucial for integrating new neurons into existing circuits to support learning, memory, and pattern separation (Oomen et al., 2014; Li et al., 2023). From the proliferation and differentiation of neural stem cells to the final development into mature dentate granule cells, each stage of AHN has its corresponding marker (Ribeiro and Xapelli, 2021). Ki-67 is a protein associated with cell proliferation and is a marker for the mitosis and proliferation of early stem cells in AHN into type 2A precursor cells (Graefe et al., 2019); DCX is a protein essential for neuronal differentiation and is often used to label type 2B precursor cells and immature neurons in the late stages of AHN (Mendez-David et al., 2023). In this study, we assessed these markers to determine the impact of ENA on AHN. Compared with the TC group, the expression of Ki-67 and DCX in the hippocampus of rats in the Mod group decreased. After electroacupuncture treatment, the levels of these two markers in the ENA group increased significantly, approaching those in the TC group. This indicates that ENA can stimulate neural stem cell proliferation and differentiation in the dentate gyrus of the hippocampus in rats with chronic sleep deprivation, promote hippocampal neurogenesis, and exert neuroprotective effects.

Synaptic plasticity is the brain’s ability to perceive, encode, and retrieve information, and it is the main functional mechanism for memory storage (Bailey et al., 2015; Goto, 2022). Newborn neurons generated during hippocampal neurogenesis exhibit high excitability and low activation thresholds, facilitating synaptic plasticity and pattern separation in the hippocampus and thereby modulating memory stability and flexibility. The decline in synaptic plasticity caused by the suppression of hippocampal neurogenesis is the direct pathological basis for learning and memory disorders (Zhang et al., 2022; Long et al., 2024). CSD can trigger a series of harmful events, including neuroinflammation, oxidative stress, and autophagy, which are detrimental to both nascent and mature neurons and can lead to synaptic plasticity disorders (Giri et al., 2024; Gao et al., 2023; Li et al., 2020; Zhou et al., 2019). Multiple studies have shown that sleep deprivation reduces the number of dendritic spines in the hippocampus and the expression of activity-dependent proteins, thereby impairing synaptic plasticity (Raven et al., 2019; Delorme et al., 2019). This study directly verified the above viewpoint through transmission electron microscopy and Golgi staining techniques. We observed severe neuronal damage in the hippocampal DG region of CSD model rats, characterized by shrunken somata, condensed nuclei, and swollen, vacuolated organelles. Furthermore, alongside pathological alterations in synaptic plasticity, reductions in synaptic vesicle numbers, thinning of the postsynaptic density, widening of the synaptic cleft, as well as immature morphology and decreased density of dendritic spines are observed. Crucially, ENA treatment significantly alleviated these pathological changes in neurons and synapses. The preservation of neuronal and synaptic integrity, increased synaptic density, and elevated mushroom-shaped spine density observed in the ENA group collectively indicate that ENA’s therapeutic efficacy likely stems from its capacity to restore synaptic plasticity and potentially reactivate neural circuits, thereby facilitating the functional reorganization of memory networks.

Although our study provides phenotypic evidence that ENA promotes hippocampal neurogenesis and restores synaptic structure, the upstream molecular mechanisms responsible for these effects remain to be elucidated. Based on existing literature, we hypothesize that ENA may exert its neuroprotective effects through one or more of the following pathways: activation of BDNF/TrkB signaling, suppression of neuroinflammation (e.g., via NF-κB inhibition or promotion of microglial M2 polarization), or upregulation of the Nrf2/ARE antioxidant pathway (Lu et al., 2020; Ma et al., 2020). In our future studies, we plan to directly test these hypotheses by employing specific pharmacological inhibitors, such as a TrkB antagonist or an NF-κB inhibitor, to determine whether blocking these pathways attenuates ENA’s neurogenic and neuroprotective effects.

Our research has the following limitations. Due to the long experiment cycle, changes in estrogen can introduce many variables that affect the experiment’s results. Research shows that there are gender differences in the response to sleep deprivation (Wright et al., 2023). Therefore, this study did not choose a mixed-gender population but used male rats as the research subjects. Studying cognitive impairments associated with sleep deprivation in female rats is also important. Another limitation of this study is the lack of an immediate post-CSD baseline control group. This prevents us from completely excluding the possibility that the observed neurogenesis and synaptic improvement might result from natural sleep recovery over time, rather than from a specific effect of ENA. However, it is necessary to point out that previous studies have shown that, without active intervention, CSD-induced hippocampal neurogenesis and synaptic deficits recover only partially and incompletely within a comparable timeframe (Mirescu et al., 2006; Wang et al., 2025). Therefore, the near-full restoration observed in the ENA group likely reflects a genuine therapeutic effect. In addition, the small sample size for the histological and ultrastructural analyses is also an important limitation of this study. Although we acknowledge this limitation, several factors support the reliability of these findings. Firstly, the results from the three independent methods are consistent with one another and agree with the behavioral results (*N* = 12). Secondly, for techniques that require extensive operations, the standard sample size for quantitative analysis in the literature is *N* = 3–4 per group, which is acceptable (Denaro et al., 2024). Importantly, *N* = 3 adheres to the Reduction principle of the 3Rs by using the minimum number of animals necessary for reliable data—a standard practice for these labor-intensive techniques (Richter, 2024). Future studies with larger sample sizes (*N* = 6–10), inclusion of an immediate post-modeling baseline group, and female rats across different recovery periods are warranted to validate and extend our findings.

## Conclusion

In male Wistar rats, ENA promotes the proliferation and differentiation of neural stem cells during hippocampal neurogenesis by upregulating the expression of DCX and Ki-67 proteins, alleviates damage to neuronal and synaptic plasticity, and improves neurocognitive function. However, it should be noted that the molecular mechanism by which ENA regulates neurogenesis and synaptic plasticity remains unclear, and the exclusive use of male rats limits the generalizability of the research results. Exploring the molecular targets of ENA during neurogenesis in the adult hippocampus and incorporating female animal models will be the focus of future research.

## Data Availability

The original contributions presented in the study are included in the article. The original images (Data Sheet 1–5) and raw data (Table 1 and Table 2) are provided as Supplementary Material for reference. Further inquiries can be directed to the corresponding author.
